# Dynamic functional evaluation of the anti-reflux mechanism with simultaneous endoscopy and manometry as a provocative test

**DOI:** 10.1055/a-2739-2127

**Published:** 2025-11-26

**Authors:** Haruhiro Inoue, Mayo Tanabe, Satoshi Abiko, Kazuki Yamamoto, Kaori Owada, Yohei Nishikawa, Stefan Seewald

**Affiliations:** 1 378609 Digestive Diseases Center, Showa Medical University Koto Toyosu Hospital, Tokyo, Japan; 2 Center for Gastroenterology, Hirslanden Clinic, Zurich, Switzerland

Comprehensive evaluation of the anti-reflux barrier remains a challenge in gastroesophageal reflux disease. This barrier involves not only the esophagogastric junction and lower esophageal sphincter (LES), but also the upper esophageal sphincter (UES), which prevents supra-esophageal reflux. Conventional endoscopy provides only structural findings, while high-resolution manometry (HRM) measures esophageal motility. The endoscopic pressure study integrated system (EPSIS 1 2 3 4 ) enables functional assessment by recording intragastric pressure and belching sound during insufflation as a provocative test.

From these observations, we proposed the phase concept 5 , describing three sequential phases: cardia closure (phase 1), LES contraction (phase 2), and esophageal peristalsis after LES relaxation (phase 3). Subsequent work highlighted a critical role of UES activity within phase 3, detectable only with HRM; thus we refined the framework into phase 3A (UES contraction after LES relaxation) and phase 3B (esophageal peristalsis after UES relaxation; Fig. 1 ).

**Fig. 1:**
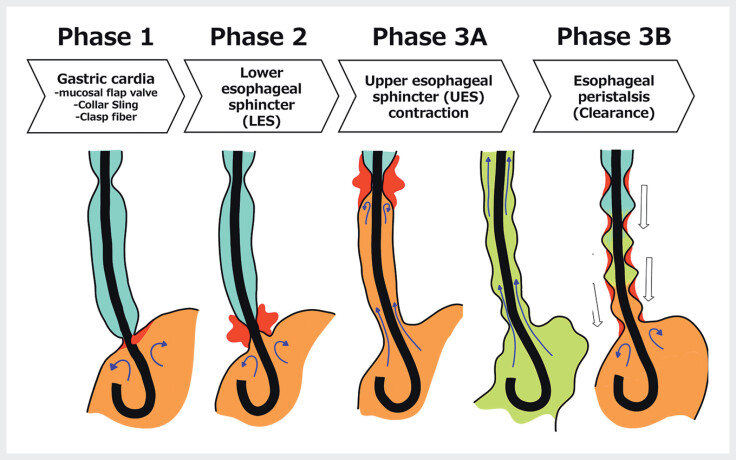
Illustration of the refined phase concept for evaluation of the anti-reflux mechanism. Phase 1: Gastric cardia closure is maintained by the mucosal flap valve, collar sling, and clasp fibers. Phase 2: Lower esophageal sphincter (LES) contraction provides further reinforcement of the barrier. Phase 3A: After LES relaxation, contraction of the upper esophageal sphincter (UES) occurs, preventing supra-esophageal reflux. Phase 3B: Following UES relaxation, esophageal body peristalsis is induced, facilitating clearance of the esophagus. Together, these sequential phases represent the coordinated physiological cycle of the anti-reflux barrier.

To operationalize this framework, we developed the endoscopic functional study integrated system (EFSIS), integrating EPSIS with HRM for simultaneous, real-time assessment of LES- and UES-related mechanisms and esophageal peristalsis. We report the first clinical application of EFSIS in the evaluation of the anti-reflux mechanism. A 35-year-old man with persistent heartburn underwent EFSIS. Endoscopy showed no esophagitis. During gastric insufflation, endoscopic images, EPSIS tracings, belching sounds, and HRM signals were recorded simultaneously ( Fig. 2 ). Phase 1 demonstrated competent cardia closure, phase 2 showed LES contraction, and phases 3A and 3B confirmed coordinated UES contraction and esophageal peristalsis, respectively ( Video 1 ). Both pH-impedance and EFSIS confirmed preserved anti-reflux barrier function. This case illustrates the first clinical application of EFSIS, demonstrating the feasibility of integrating EPSIS with HRM to dynamically visualize and quantify anti-reflux mechanisms, including LES, UES, and esophageal peristalsis. This novel multimodal approach may provide complementary insights into reflux pathophysiology and has potential as a diagnostic tool in patients with reflux symptoms.

**Fig. 2:**
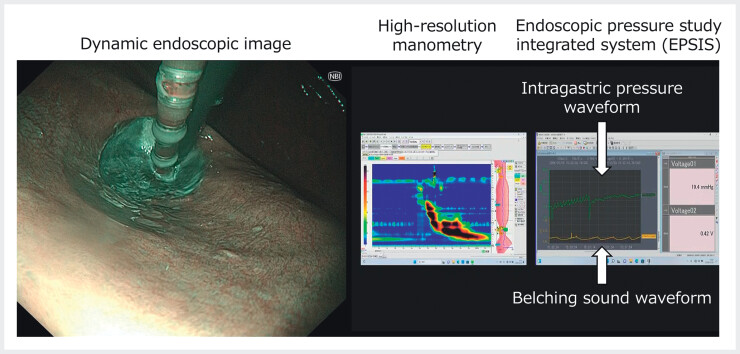
Endoscopic functional study integrated system (EFSIS). Dynamic endoscopic imaging is combined with high-resolution manometry, intragastric pressure monitoring, and belching sound recording (EPSIS). The system enables the simultaneous evaluation of endoscopic findings, intragastric pressure waveforms, belching sound waveforms, and esophageal motility in real time.

This video demonstrates the dynamic evaluation of the anti-reflux mechanism using the endoscopic functional study integrated system (EFSIS). During gastric insufflation, simultaneous recordings of endoscopy, intragastric pressure, belching sound, and high-resolution manometry were obtained.Video 1

Endoscopy_UCTN_Code_CCL_1AB_2AC_3AC

## Notes

Endoscopy E-Videos https://eref.thieme.de/e-videos
*E-Videos* is an open access online section of the journal *Endoscopy* , reporting on interesting cases and new techniques in gastroenterological endoscopy. All papers include a high-quality video and are published with a Creative Commons CC-BY license. Endoscopy E-Videos qualify for HINARI discounts and waivers and eligibility is automatically checked during the submission process. We grant 100% waivers to articles whose corresponding authors are based in Group A countries and 50% waivers to those who are based in Group B countries as classified by Research4Life (see: https://www.research4life.org/access/eligibility/ ). This section has its own submission website at https://mc.manuscriptcentral.com/e-videos .
